# Clinical benefits of PD-1/PD-L1 inhibitors in patients with metastatic colorectal cancer: a systematic review and meta-analysis

**DOI:** 10.1186/s12957-022-02549-7

**Published:** 2022-03-24

**Authors:** Xiao Zhang, Zhengyang Yang, Yongbo An, Yishan Liu, Qi Wei, Fengming Xu, Hongwei Yao, Zhongtao Zhang

**Affiliations:** Department of General Surgery, Beijing Friendship Hospital, Capital Medical University, 95 Yong-an Rd, Xi-Cheng District, Beijing, China

**Keywords:** Immune checkpoint inhibitors, Colorectal neoplasms, Meta-analysis, Clinical efficacy

## Abstract

**Background:**

Immunotherapy for colorectal cancer has developed rapidly in the past decade. Many high-quality clinical trials examining the application of PD-1/PD-L1 inhibitors in patients with metastatic colorectal cancer (mCRC) have been conducted in recent years. However, the clinical benefits, including the efficacy and safety of these treatments against mCRC, remain controversial. Hence, we conducted this meta-analysis on the clinical benefits of PD-1/PD-L1 inhibitors in patients with mCRC.

**Methods:**

We searched online databases including MEDLINE, Embase, Cochrane Library, and Web of Science, from inception to January 4, 2021. The outcomes related to efficacy and safety were extracted and analyzed. Subgroup analyses were conducted according to the categories of dMMR-MSI-H (tumors with mismatch repair deficiency and high levels of microsatellite instability) ≥ 5% vs. dMMR-MSI-H < 5%, monotherapy vs. combination therapy, PD-1 inhibitors vs. PD-L1 inhibitors, and nivolumab vs. pembrolizumab.

**Results:**

Fourteen studies including 1129 subjects were included in our systematic review. The overall complete response (CR), partial response (PR), stable disease (SD), and progression of disease (PD) rates were 0.01 (95% CI 0.00–0.04), 0.04 (95% CI 0.05–0.26), 0.27 (95% CI 0.22–0.32), and 0.44 (95% CI 0.30–0.58), respectively. The overall objective response rate (ORR) and disease control rate (DCR) were 0.16 (95%CI 0.06–0.31) and 0.50 (95%CI 0.35–0.65), respectively. The overall rate of adverse events (AEs) and severe adverse responses (SAEs) were 0.84 (95% CI 0.72–0.92) and 0.30 (95% CI 0.20–0.41), respectively. The ORRs of the dMMR-MSI-H ≥ 5% and dMMR-MSI-H < 5% subgroups were 0.40 (95% CI 0.30–0.51) and 0.04 (95% CI 0.00–0.09), respectively.

**Conclusions:**

PD-1/PD-L1 inhibitors produced encouraging clinical benefits including the response rate in the treatment of dMMR-MSI-H mCRC. They actually have been influenced by the present state of mCRC therapy including pMMR-MSI-L mCRC. Nevertheless, additional multi-center prospective studies are still expected.

**Trial registration:**

We have registered this study in the International Prospective Register of Systematic Reviews (PROSPERO), and the registration number is CRD42021249601.

**Supplementary Information:**

The online version contains supplementary material available at 10.1186/s12957-022-02549-7.

## Background

Colorectal cancer (CRC) is the third most common cancer, accounting for 10.2% of diagnosed cancers annually [1, 2]. Localized colorectal cancer can be treated with curative surgery followed by chemotherapy with a favorable prognosis. However, a large proportion of people are initially diagnosed with metastatic CRC (mCRC) because early-stage CRC may be asymptomatic [3]. Unfortunately, current therapies are unable to achieve good therapeutic effects, further resulting in a poor prognosis for most patients with mCRC [4]. The 5-year survival rate is only approximately 14% [5]. In the past decade, immunotherapy has developed rapidly and attracted increasing attention because of its excellent antitumor effect when used in clinical applications, which provides power and hope for patients with advanced cancer and mCRC. Immunotherapy kills cancer cells by activating human antitumor immunity relative to traditional therapies. In addition, immunotherapy always specifically targets cancer antigens, preventing normal cells from being attacked. In some cases, immunotherapy has yielded promising results. Therefore, immunotherapy may be a new alternative treatment for mCRC [6–8].

**Fig. 1:**
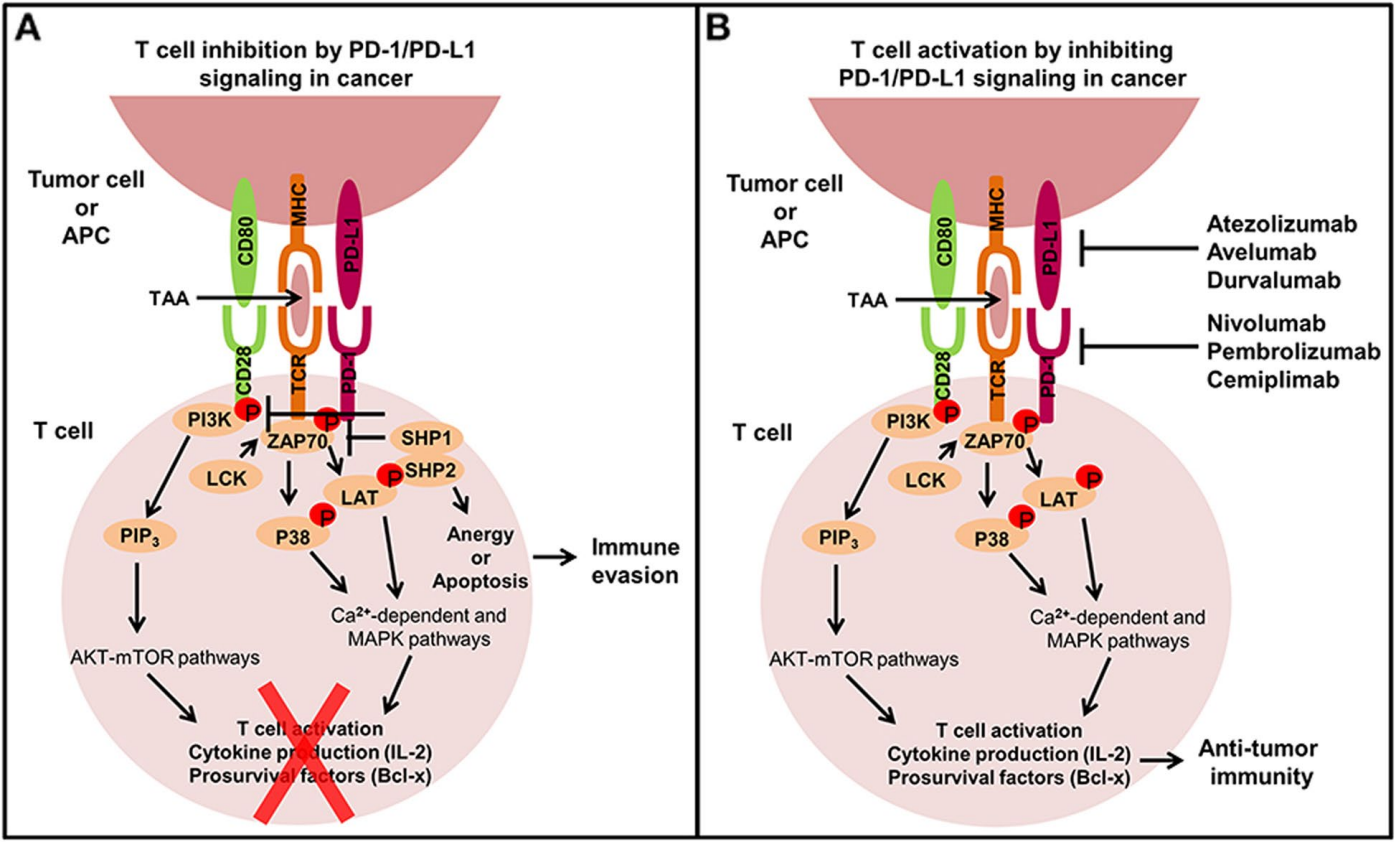
The extrinsic function of the PD-1/PD-L1 signaling axis in cancer and the mechanisms of PD-1/PD-L1 inhibitors in cancer therapy. **A** When the overexpressed PD-L1 of tumors bind to the PD-1 expressed on the T cells, it will lead to T cells anergy or apoptosis and ultimately immune evasion. **B** PD-1/PD-L1 inhibitors can block this pathway to promote T cell activation [11]. Copyright © 2020 Hudson, Cross, Jordan-Mahy, and Leyland

Meanwhile, programmed death 1 (PD-1) is a key immune-checkpoint receptor expressed by activated T cells, and it mediates immunosuppression, while membrane-bound programmed death 1 ligand 1 (PD-L1) engages programmed death 1, leading to T cell anergy and/or apoptosis [9, 10]. Thus, PD-1/PD-L1 inhibitors prevent T cell dysfunction and apoptosis and instead enhance T cell activation, providing a new choice for the treatment of cancer (Fig. 1) [11]. Since the first use of nivolumab in humans in 2006, many clinical trials using PD-1/PD-L1 inhibitors for the treatment of various refractory cancers such as melanoma and lung cancer have been conducted [12]. A number of trials have shown that PD-1/PD-L1 inhibitors result in a survival benefit. Currently, five FDA-approved PD-1/PD-L1 inhibitors are used in cancer therapeutics: nivolumab, pembrolizumab, atezolizumab, durvalumab, and avelumab [13].

In mCRC, T cell infiltration into the tumor bed has long been associated with favorable outcomes, suggesting that PD-1/PD-L1 inhibitors may be effective against mCRC [9]. According to the mutation pattern, CRC is categorized into two groups: tumors with mismatch-repair deficiency and high levels of microsatellite instability (dMMR-MSI-H CRC), and tumors with mismatch repair proficient and low levels of microsatellite instability (pMMR-MSI-L CRC) [14, 15]. dMMR-MSI-H CRC accounts for approximately 15% of colorectal cancer cases and approximately 4–5% of patients with mCRC have this type of tumor [15–18]. Many clinical trials have proven that dMMR-MSI-H CRC might be more sensitive to treatment with immune checkpoint inhibitors (ICIs) including PD-1/PD-L1 inhibitors. Therefore, immune checkpoint therapy is approved as a treatment for dMMR-MSI-H CRC by the Food and Drug Administration [16, 17, 19].

Nevertheless, due to the lower proportion of MSI-H-dMMR mCRC, PD-1/PD-L1 inhibitors have been used in combination with adjuvant therapy for better treatment efficacy against mCRC in different clinical trials. Therefore, the clinical benefits, including the efficacy and safety, of these treatments against mCRC remain controversial. Hence, we thoroughly searched the relevant literature and conducted this systematic review and meta-analysis to assess the use of PD-1/PD-L1 inhibitors in patients with mCRC and to reduce the deficiencies of individual studies and estimate the overall benefits.

## Methods

### Data source and search strategy

Clinical trials using PD-1/PD-L1 inhibitors as part of primary treatment for adult patients with mCRCs were eligible for inclusion (from inception to January 4, 2021). The databases include MEDLINE, Embase, Cochrane Library, and Web of Science. The search terms used to define the therapy were “immune checkpoint inhibitors,” “PD-L1 inhibitors,” “CTLA-4 inhibitors,” “PD-1 inhibitors,” “PD-1-PD-L1 Blockade,” “nivolumab,” “atezolizumab,” “durvalumab,” “avelumab,” and “pembrolizumab.” The search terms used to define the disease were “colorectal neoplasms,” “colorectal cancer,” “colorectal carcinoma,” and “colorectal tumor.” For example, the search query in PubMed was “((((((((((Immune Checkpoint Inhibitors [MeSH Terms]) OR (PD-L1 Inhibitors)) OR (CTLA-4 Inhibitors)) OR (PD-1 Inhibitors)) OR (PD-1-PD-L1 Blockade)) OR (nivolumab)) OR (pembrolizumab)) OR (atezolizumab)) OR (durvalumab)) OR (avelumab)) AND ((((Colorectal Neoplasms [MeSH Terms]) OR (colorectal cancer)) OR (Colorectal Carcinoma)) OR (Colorectal Tumours))”. Additionally, the reference lists of all relevant articles were checked to avoid omissions. Two authors (Xiao Zhang and Zhengyang Yang) independently searched the databases and independently reviewed the extracted studies. We have registered this study in the International Prospective Register of Systematic Reviews (PROSPERO), and the registration number is CRD42021249601.

### Study selection criteria

**Fig. 2:**
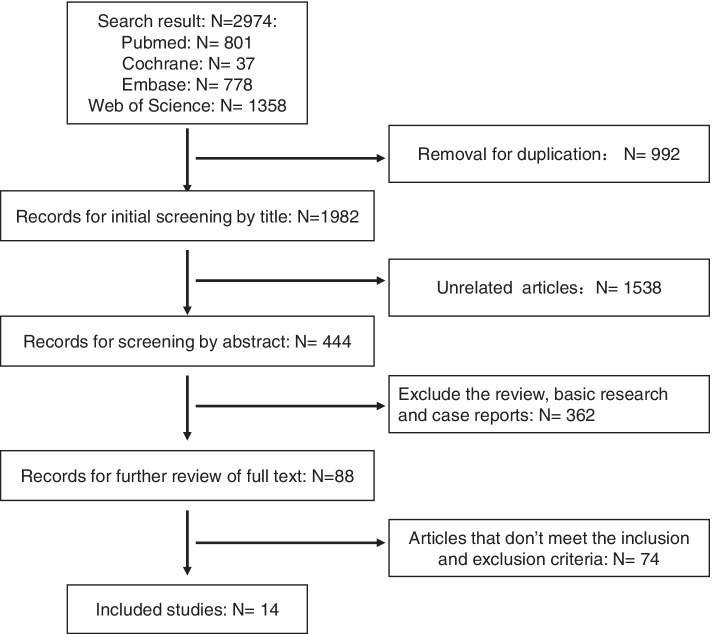
Flow chart of study selection

The inclusion criteria are described below. (1) The subjects were patients with diagnosed with mCRC, which was also described as “unresectable CRC,” “advanced CRC,” “first-line treatment failure CRC,” etc. For the studies not exclusively including patients with mCRC, as long as they provided the detailed data of these patients, they were included in the analysis. (2) The interventions were monotherapy with PD-1/PD-L1 inhibitors or combination treatment with other drugs. (3) The study focused on the efficacy of PD-1/PD-L1 inhibitors, and the clinical data, such as the objective response rate (ORR), disease control rate (DCR), complete response (CR), partial response (PR), stable disease (SD), progression of disease (PD), adverse events (AEs), and severe adverse events (SAEs), were all provided in detail or were able to be calculated. (4) The MSI/MMR state of patients was described clearly. (5) The articles were published in English, and the study types were clinical trials, retrospective studies, prospective studies, or case series. The exclusion criteria were as follows: (1) studies that did not exclusively include patients with mCRC and we were unable to obtain data from patients with mCRC from the paper; (2) patients treated with PD-1/PD-L1 inhibitors combined with radiotherapy; (3) early publications from studies analyzing the same group of patients; and (4) publications such as letters, reviews, case reports, protocols, or editorial articles (Fig. 2).

### Efficacy indicators

The main outcome measures included ORR, DCR, CR, PR, SD, PD, AEs, SAEs, 1-year overall survival rate (OS), and 1-year progression-free survival rate (PFS). CR, PR, SD, and PD were all assessed according to Response Evaluation Criteria In Solid Tumors (RECIST), version 1.1. ORR refers to the proportion of patients who had a confirmed objective response of complete or partial response. In other words, ORR is the sum of CR and PR. Similarly, DCR is the sum of ORR and SD. Moreover, SAEs are defined as adverse events that are grade 3 or higher according to the Common Terminology Criteria for Adverse Events (CTCAE).

### Data extraction

Two investigators skimmed the full text of the selected studies and extracted the necessary data independently. The first author’s name and the year of publication were used together to identify the study. As shown in Table 1, the information we extracted included the following items: first author’s name, year of publication, trial phase, journal of publication, interventions (type and dose of PD-1/PD-L1 inhibitor and combination therapy), number, median age and male/female ratio of the subjects, the dMMR-MSI-H ratio of the subjects, median follow-up time, and outcomes (OS, PFS, AEs, SAEs, ORR, CR, etc.). All of the extracted data were recorded in an Excel spreadsheet. Any differences were resolved by discussion until consensus was reached or by consulting the corresponding author (Zhongtao Zhang and Hongwei Yao). If data on the main outcomes were missing, we contacted the investigators.

**Table 1:** Brief characteristics of included studies

First author	Year	Journal	Study stage	No. of patients^a^	PD-1/PD-L1 inhibitors	Dose	Combination drug	Male (%)	Median age	dMMR-MSI-H rate (%)	Median follow-up time
M. J. Overman	2017	Lancet Oncol	II	74	Nivolumab	3 mg/kg iv q2w	None	44 (59)	52.5	100 %	12m
B. H. O'Neil	2017	PLoS One	Ib	23	Pembrolizumab	10mg/kg iv q2w	None	13 (57)	57	4 %	5.3m
M. D. Hellmann	2019	Ann Oncol	Ib	84	Atezolizumab	800 mg iv q2w	Cobimetinib	N/A	N/A	2.30 %	17m
D. T. Le	2019	J Clin Oncol	II	124	Pembrolizumab	200 mg q3w	None	69 (56)	56	100 %	31.3m/24.2m ^b^
T. André	2020	N Engl J Med	III	153	Pembrolizumab	200mg q3w	None	71 (46)	63	100 %	32.4m
D.T. Le	2015	N Engl J Med	II	28	Pembrolizumab	10mg/kg iv q2w	None	N/A	N/A	36 %	9m/5m ^b^
M. J. Overman	2018	J Clin Oncol	II	119	Nivolumab	3mg/kg iv q3w/q2w	Ipilimumab	70 (59)	58	100 %	13.4m
E. X. Chen	2020	JAMA Oncol	II	119	Durvalumab	1500mg iv q4w	Tremelimumab	74 (62)	65	<2 %	15.2m
R. Cohen	2020	J Immunother Cancer	II	57	Nivolumab	3mg/kg iv q3w/q2w	Ipilimumab	30 (53)	56.5	100 %	12m
C. Wang^c^	2020	Oncologist	-	18	Nivolumab/ pembrolizumab	N:240mg iv q2w, P:200mg iv q3w	Regorafenib	16 (89)	60	0 %	7m
S. Fukuoka	2020	J Clin Oncol	Ib	25	Nivolumab	3mg/kg q2w	Regorafenib	18 (72)	55	4 %	N/A
J. Li^c^	2020	Front Oncol	-	23	Different kinds of inhibitors	&	Regorafenib	16 (70)	50	0 %	7.9m
C. Ren	2020	Am J Cancer Res	II	9	Shr-1210	200mg iv q2w	Apatinib	N/A	54	0 %	N/A
C. Eng	2019	Lancet Oncol	III	273	Atezolizumab	840mg iv q2w/ 1200mg iv q3w	Cobimetinib or none	166 (61)	56	2.20 %	7.3m

*N/A* the data is not mentioned in the paper or we cannot get the accurate number due to the withdrawal of participants or other reasons

^a^This data is the number of patients who are enrolled in the final analysis of the study

^b^In these studies, the patients are divided into two cohorts with different median follow-up time

^c^These studies are retrospective. & nivolumab:240mg q2w; camrelizumab 200mg q2/3w; toripalimab 240mg q3w; pembrolizumab/sintilimab 200mg q3w

### Quality assessment

Two investigators independently assessed the quality of the included studies with the “IHE quality appraisal checklist for assessing case-series studies” reported in the study by Guo et al. [20]. The checklist contains 20 questions rating various aspects of the studies, including study objective, study design, study population, intervention and cointerventions, outcome measures, statistical analysis, the results and conclusions, competing interests, and sources of support.

### Statistical analysis

The meta-analysis was performed using Stata/MP 14.0 software (StataCorp LP, College Station, TX). A *p* value < 0.05 was considered statistically significant. Because of the characteristics of the data from a single-arm study, we performed a double arcsine transformation. We used the *I*^2^ test to quantify the heterogeneity of the studies. The studies were considered to have substantial heterogeneity if *I*^2^≥50%. Because of the high heterogeneity of the studies, we selected random-effect models for all meta-analyses. Meta-regression analysis was used to explore the source of heterogeneity. If *p*<0.05, we presumed that this factor was related to heterogeneity. We conducted subgroup analyses based on clinical factors to reduce heterogeneity. The main stratification factor was the MSI-H/dMMR status. dMMR-MSI-H rate is defined as the ratio of the number of patients with dMMR-MSI-H to the total number of patients enrolled in the study. According to the proportion of patients with dMMR-MSI-H colorectal cancer in each study, we divided the studies into the dMMR-MSI-H ≥ 5% subgroup and dMMR-MSI-H < 5% subgroup and conducted a subgroup analysis. Supplementary stratification factors included the type of therapy (monotherapy vs. combination therapy), the drug target (PD-1 vs. PD-L1), and the type of PD-1 inhibitor (pembrolizumab vs. nivolumab). Publication bias was evaluated using Egger’s test

## Results

### Article selection and characteristics of the included studies

The initial search retrieved 2974 articles, including 801 articles from MEDLINE, 778 articles from Embase, 37 articles from the Cochrane Library, and 1358 articles from Web of Science. After removal of the duplicate articles using EndNote and manual methods, 1982 articles remained. Then, two authors skimmed the titles of these articles and excluded 1538 articles that were unrelated to our theme. After reviewing the abstracts, we removed the reviews, basic research articles, and case reports. Eighty-eight articles were retained for the full-text review. As shown in Fig. 2, 14 studies including 1129 subjects were included in our meta-analysis according to the inclusion criteria [17, 19, 21–32]

Among the 14 studies, 3 were multi-arm studies, and the rest were single-arm studies. For multi-arm studies, we focused on the use of PD-1/PD-L1 inhibitors. Additionally, 2 retrospective studies were included in the selected studies. All were published in the last 6 years because of the development of immune checkpoint inhibitors for CRC. In addition, the article “C. Eng 2019” contained two arms, including monotherapy and combination therapy, and thus, we separated the two groups for analysis. Six groups adopted monotherapy, and nine adopted combination therapy combined with various drugs, such as cobimetinib and regorafenib. Eleven and three studies focused on PD-1 inhibitors and PD-L1 inhibitors, respectively. Among the 11 studies that used PD-1 inhibitors, 5 studies including 293 patients adopted nivolumab, while 4 studies including 327 patients adopted pembrolizumab. Of the studies that provided clear data, the proportion of males was 58%, and the age range was 21–93 years (Table 1).

### Quality assessment and analysis of publication bias

**Fig. 3:**
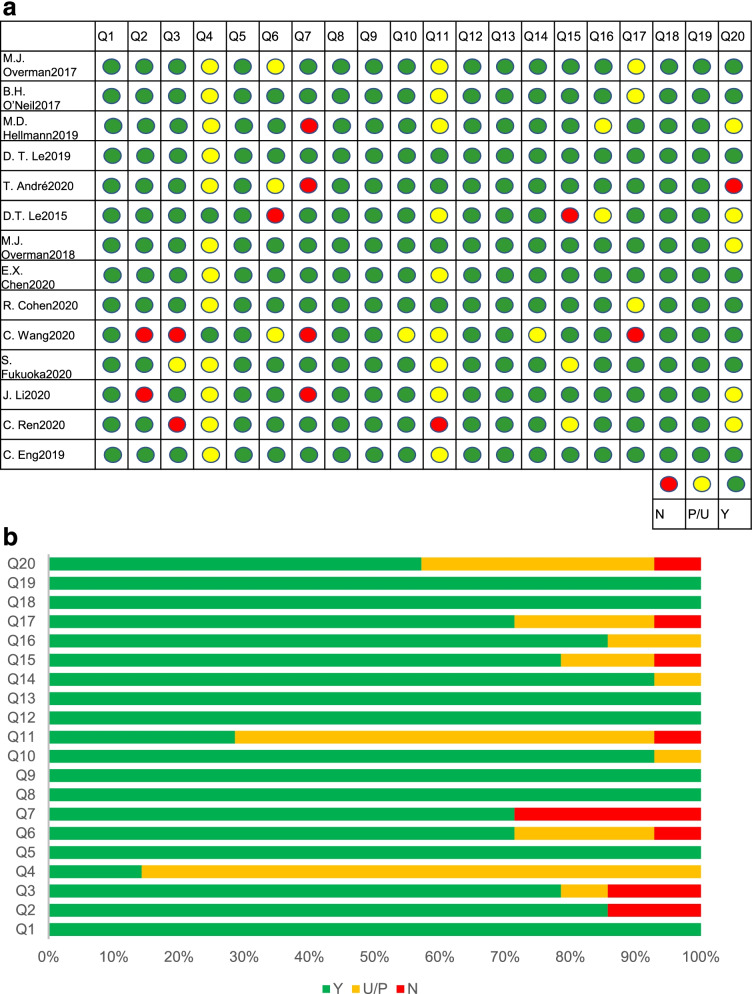
Quality Assessment of included articles. This figure shows the result of assessing the included articles by “IHE’s quality appraisal checklist for assessing case-series studies.” **A** The detailed results of all 14 studies for the answers to the 20 questions in the checklist. **B** The distribution of answers for each question in the checklist. Y yes, *U/P* unclear/partial, *N* no

Using the methods mentioned above, we assessed all 14 articles and combined them in Fig. 3. For these questions, we assigned 1 point if the answer was “yes” and assigned 0 points if the answer was “Unclear/Partial” or “No.” Using this approach, the highest score was 20. All studies received at least 12 points. We did not identify statistically significant publication bias using Egger’s test (Table S1).

### Meta-regression analysis

Due to the high heterogeneity in the meta-analysis of single-arm studies, we conducted a meta-regression analysis to explore the source of heterogeneity. The MSI status is associated with the heterogeneity of ORR, DCR, CR, PR, PD, PFS, and OS. The choice of monotherapy or combination therapy is only associated with the heterogeneity of DCR. The choice of PD-1/PD-L1 inhibitors is associated with the heterogeneity of CR, AEs, and OS. The type of PD-1 inhibitor was associated with the heterogeneity of ORR, PR, SD, and PFS (Table 2).

**Table 2:** Meta regression

	MSI status	Monotherapy or combination therapy	PD-1 or PD-L1 inhibitors	Nivolumab, pembrolizumab or others (Nivolumab)	Nivolumab, pembrolizumab or others (pembrolizumab)
ORR	0*	0.576	0.086	0.079	0.017*
DCR	0.012*	0.04*	0.688	0.062	0.964
CR	0.001*	0.566	0.017*	0.821	0.406
PR	0.001*	0.567	0.076	0.044*	0.009*
SD	0.835	0.284	0.335*	0.084	0.018*
PD	0.004*	0.972	0.051	0.1	0.549
AEs	0.607	0.259	0.036*	0.44	0.867
SAE	0.51	0.151	0.058	0.958	0.606
PFS	0.034*	0.75	0.908	0.031*	0.122
OS	0.029*	0.962	0.025*	0.188	0.188

*P* value < 0.05 means significant statistical differences. *ORR* objective response rate, *DCR* disease control rate, *CR* complete response rate, *PR* partial response rate, *SD* stable disease rate, *PD* progression disease rate, *AEs* adverse events, *SAEs* severe adverse events, *PFS* 1-year progression-free survival rate, *OS* 1-year overall survival rate (OS)

### Subgroup analysis of the response rate

All 14 eligible studies provided response rates (CR, PR, SD, and PD) directly or indirectly. As shown in Table 3, for all 1129 patients treated with PD-1/PD-L1 inhibitors, CR was achieved in 41/1129 subjects (0.01, 95% CI 0.00–0.04), PR was achieved in 219/1129 subjects (0.04, 95% CI 0.05–0.26), SD was achieved in 284/1129 subjects (0.27, 95% CI 0.22–0.32), and PD was achieved in 513/1129 patients (0.44, 95% CI 0.30–0.58). The ORR and DCR were calculated from the response rate data. The overall ORR was 0.16 (95% CI 0.06–0.31), and the DCR was 0.50 (95% CI 0.35–0.65). However, the heterogeneity of the studies was high.

**Table 3:** Overall response rate of included studies

	Number	Rate	95%CI	*I*^2^%
ORR	260/1129	0.16	0.06–0.31	96.39
DCR	544/1129	0.5	0.35–0.65	95.57
CR	41/1129	0.01	0.00–0.04	82.95
PR	219/1129	0.14	0.05–0.26	95.20
SD	284/1129	0.27	0.22–0.32	66.95
PD	513/1129	0.44	0.30–0.58	95.09

*ORR* objective response rate, *DCR* disease control rate, *CR* complete response rate, *PR* partial response rate, *SD* stable disease rate, *PD* progression disease rate (PD)

**Fig. 4:**
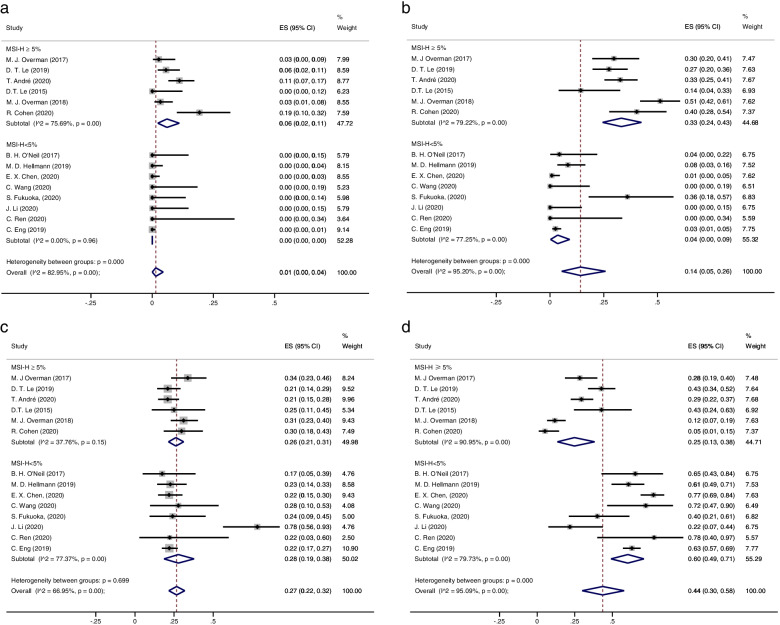
The forest figure of response rate (CR, PR, SD, PD) on MMR-MSI subgroup analysis. **A** CR rate on MMR-MSI subgroup analysis. **B** PR rate on MMR-MSI subgroup analysis. **C** SD rate on MMR-MSI subgroup analysis. **D** PD rate on MMR-MSI subgroup analysis. Complete response rate (CR), partial response rate (PR), stable disease rate (SD), progression disease rate (PD)

In the subgroup analysis of the dMMR-MSI-H ≥ 5% and dMMR-MSI-H < 5% groups, 41/555 patients (0.06, 95% CI 0.02–0.11) vs. 0/574 patients (0.00, 95% CI 0.00–0.00) achieved CR, respectively; PR was achieved in 194/555 (0.33, 95% CI 0.24–0.43) vs. 25/574 (0.04, 95% CI 0.00–0.09); SD was achieved in 144/555 patients (0.26, 95% CI 0.21–0.31) vs. 140/574 patients (0.28, 95% CI 0.19–0.38); and PD was achieved in 148/555 patients (0.25, 95% CI 0.13–0.38) vs. 365/574 patients (0.60, 95% CI 0.49–0.71), respectively (Fig. 4). For the monotherapy and combination therapy groups, CR was achieved in 26/492 patients (0.02, 95% CI 0.00–0.07) vs. 15/637 patients (0.01, 95% CI 0.00–0.04); PR was achieved in 113/492 patients (0.17, 95% CI 0.07–0.31) vs. 106/637 (0.11. 95% CI 0.01–0.27); SD was achieved in 111/492 patients (0.22 95% CI 0.18–0.27) vs. 173/637 (0.29 95% CI 0.22–0.38); and PD was achieved in 207/492 patients (0.45, 95% CI 0.31–0.59) vs. 306/637 patients (0.45, 95% CI 0.24–0.67) (Table S2, Fig. S1). For the analysis of PD-1 and PD-L1 therapy groups, 41/644 patients (0.03, 95% CI 0.01–0.07) vs. 0/476 patients (0, 95% CI 0.00–0.00) achieved a CR; 201/644 patients (0.20 95% CI 0.11–0.31) vs. 15/476 patients (0.03, 95% CI 0.01–0.07) achieved a PR; 179/644 patients (0.29, 95% CI 0.22–0.37) vs. 105/476 patients (0.22, 95% CI 0.18–0.26) achieved SD; and 198/644 patients (0.36, 95% CI 0.24–0.49) vs. 315/476 patients (0.67, 95% CI 0.57–0.77) achieved PD (Table S3, Fig. S2).

**Fig. 5:**
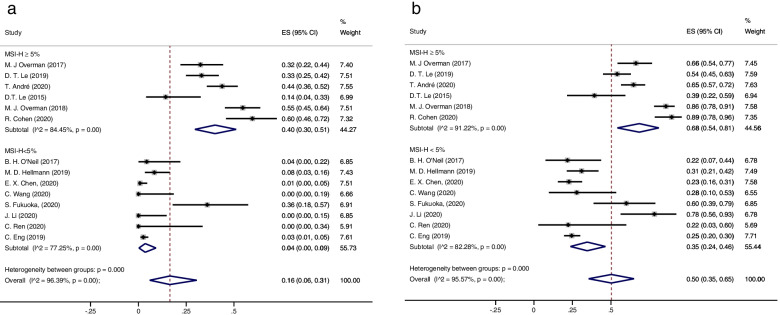
The forest figure of response rate (ORR, DCR) on MMR-MSI subgroup analysis. **A** ORR rate on MMR-MSI subgroup analysis. **B** DCR rate on MMR-MSI subgroup analysis. Objective response rate (ORR), disease control rate (DCR)

In the subgroup analysis, the ORR and DCR were 0.40 (95% CI 0.30–0.51) and 0.68 (0.54–0.81) in the dMMR-MSI-H ≥ 5% group and 0.04 (95% CI 0.00–0.09) and 0.35 (95% CI 0.24–0.46) in the dMMR-MSI-H < 5% group, respectively (Fig. 5). For the analysis of monotherapy and combination therapy groups, the ORRs were 0.20 (95% CI 0.07–0.37) and 0.12 (95% CI 0.01–0.31), while the DCRs were 0.45 (95% CI 0.29–0.62) and 0.51 (95% CI 0.29–0.72), respectively. In addition, in the PD-1 group, the ORR and DCR were 0.23 (95% CI 0.12–0.36) and 0.59 (95% CI 0.45–0.71), respectively, while in the PD-L1 group, the values were 0.03 (95% CI 0.01–0.07) and 0.25 (95% CI 0.21–0.29), respectively (Table S2, Table S3).

### Subgroup analysis of the prognosis

In all 14 papers, the authors chose different indicators to evaluate the survival status. The median PFS and OS times were not reached or were unavailable in many studies. Hence, we chose the 1-year PFS rate and OS rate for analysis. Nine papers reported the 1-year PFS rate, and 7 papers reported the 1-year PFS rate. For other papers that did not report the relevant data, we emailed the corresponding author but received no response. The overall 1-year PFS rate and OS rate were 0.40 (0.26–0.55) and 0.79 (0.72–0.85), respectively. The subgroup analysis of these two indicators is presented in Table 4.

**Table 4:** Subgroup analysis on prognosis

	No. of studies	Rate	95%CI	*I*^2^%	No. of studies	Rate	95%CI	*I*^2^%^a^
dMMR-MSI-H ≥ 5%					dMMR-MSI-H<5%			
OS	4	0.79	0.72-0.85	54.96	3	0.34	0.05-0.72	-
PFS	5	0.57	0.44-0.69	88.85	4	0.17	0.01-0.46	92.19
PD-1					PD-L1			
OS	6	0.72	0.6-0.82	82.7	1	0.11	0.05-0.19	-
PFS	8	0.39	0.23-0.56	93.87	1	0.43	0.32-0.54	-
Monotherapy					Combination therapy			
OS	3	0.62	0.41-0.80	-	4	0.62	0.2-0.95	98.03
PFS	4	0.37	0.21-0.54	90.64	5	0.43	0.19-0.68	94.78

^a^*I*^2^ cannot be calculated because the number of studies is 3 or less than 3. One-year overall survival rate (OS), one-year progression-free survival rate (PFS)

### Subgroup analysis of adverse events

All 14 papers provided data on the rates of AEs and SAEs. Among all 1129 patients, AEs and SAEs occurred in 945/1129 patients (0.84, 95% CI 0.72–0.92) and 402/1129 patients (0.30, 95% CI 0.20–0.41), respectively. In the subgroup analysis of monotherapy vs. combination therapy, the rate of AEs was 0.76 (95% CI 0.62–0.88) vs. 0.90 (95% CI 0.77–0.98), while the rate of SAEs was 0.21 (95% CI 0.14–0.29) vs. 0.38 (95% CI 0.25–0.53). Among the 653 patients treated with PD-1 inhibitors, AEs and SAEs occurred in 481/653 (0.76 95% CI 0.66–0.86) and 153/653 patients (0.23, 95% CI 0.15–0.32), respectively. Among the 476 patients treated with PD-L1 inhibitors, AEs and SAEs occurred in 464/476 (0.98 95% CI 0.95–1.00) and 249/653 patients (0.52, 95% CI 0.42–0.62), respectively. In the subgroup analysis of dMMR-MSI-H ≥ 5% and dMMR-MSI-H < 5%, the rate of AEs was 0.78 (95% CI 0.70–0.85) vs. 0.87 (95% CI 0.70–0.98), while the rate of SAEs was 0.25 (95% CI 0.18–0.32) vs. 0.34 (95% CI 0.19–0.51) (Table S4).

### Subgroup analysis of the PD-1-treated group

Of all 14 studies, 11 studied treated patients with different PD-1 inhibitors. The PD-1 inhibitors mainly included nivolumab and pembrolizumab. Therefore, we divided the 11 studies into 3 groups: “nivolumab group,” “pembrolizumab group,” and “other group.” If the intervention was nivolumab or pembrolizumab, the study was also assigned to the “other group.” In the 4 studies including 275 patients who received nivolumab, 17 patients (0.05, 95% CI 0.00–0.13) achieved a CR, and 115 patients (0.40, 95% CI 0.29–0.51) achieved a PR (Table S5). In the pembrolizumab group, 24 patients (0.04, 95% CI 0.00–0.10) achieved a CR, and 89 patients (0.21, 95% CI 0.11–0.33) achieved a PR. In the “other group,” 0 patients (0.00, 95% CI 0.00–0.04) achieved a CR and PR.

## Discussion

PD-1/PD-L1 inhibitors represent a new direction for the treatment of mCRC. With the FDA approval of PD-1 inhibitors for some gastrointestinal cancers [12], an increasing number of clinical trials on the use of PD-1/PD-L1 inhibitors have been conducted in the area of colorectal cancer. In our meta-analysis, we used different terms on “colorectal cancer” and “immune checkpoint inhibitors” to search online databases to prevent omitting relevant studies. Moreover, most of the 14 chosen articles used PD-1 inhibitors to treat mCRC, indicating that in recent years, researchers have become more interested in the use of PD-1 inhibitors rather than PD-L1 inhibitors.

When we conducted a subgroup analysis on the MSI-MMR status, many studies included both patients with pMMR-MSI-L and dMMR-MSI-H tumors. Therefore, we divided the studies into two groups according to the proportion of patients with dMMR-MSI-H colorectal cancer: dMMR-MSI-H ≥ 5% and dMMR-MSI-H < 5%. We chose 5% as the threshold because patients with MSI-H/dMMR CRC account for less than 5% of patients with mCRC in the real world according to a previous report [18].

We assessed the quality of the studies with the “IHE quality appraisal checklist for assessing case-series studies” reported in the study by Bing Guo et al. Currently, many well-developed methodologies are available to assess the quality of randomized control trials and nonrandomized controlled studies. However, for case-series studies, few choices are available. The checklist we chose assessed different aspects of the paper, although the disadvantage is that no official cutoff score is available to judge the quality. According to some previous systematic reviews [33, 34], we assumed that all the problems had the same weight and assigned a score of 1 to the paper when the answer was “yes” in this meta-analysis. However, currently, no standard method has been developed to score the studies with this checklist, and it has been used in various systematic reviews in different ways [35, 36].

Some multi-arm studies were included and analyzed together with other single-arm studies in this meta-analysis because the subjects in these studies also met our requirements. For example, in the study by Eng (2019), the author divided the patients into three cohorts and administered different treatments: atezolizumab combined with cobimetinib, atezolizumab monotherapy, and regorafenib monotherapy. During the meta-analysis, we ignored the regorafenib group and merged the first two groups. When conducting the subgroup analysis of monotherapy vs. combination therapy, we analyzed the first two groups. Using this approach, we included more studies to ensure that this meta-analysis was more rigorous.

High heterogeneity existed in our meta-analysis of single-arm studies, and thus, we performed meta-regression and subgroup analyses to solve this problem. Similar to the results of most studies, the MMR-MSI status is obviously related to the effectiveness (ORR, DCR, CR, PR, and PD) of PD-1/PD-L1 inhibitors and survival data (PFS and OS) because dMMR-MSI-H tumors significantly upregulate immune checkpoint proteins such as PD-1/PD-L1 and exhibit increased sensitivity to PD-1/PD-L1 inhibitors, which has been reported in other papers [37]. In the forest maps of ORR and PR, two studies were significantly different from other studies in the same subgroup.

In the dMMR-MSI-H ≥ 5% subgroup, the study by Le et al. showed obviously lower ORR and PR values than other studies. We propose that the possible explanation is that this study included fewer patients with dMMR-MSI-H colorectal cancer. The ORR and PR of the dMMR-MSI-H < 5% subgroup in the study by Fukuoka et al. [29] were both 0.36 (95% CI 0.18–0.57), and the PD and DCR were 0.60 (95% CI 0.39–0.79) and 0.40 (0.21–0.61), respectively, which were significantly different than other studies in this subgroup analysis. This result is very encouraging regarding the use of PD-1/PD-L1 inhibitors for patients with pMMR-MSI-L tumors.

Additionally, two retrospective studies (Li et al. [30] and Wang et al. [28]) included in this analysis also chose PD-1 inhibitors combined with regorafenib as a therapeutic schedule, similar to the study by Fukuoka et al. [29]. Li et al. [30] and Wang et al. [28] did not achieve similar ORRs and PRs as Fukuoka et al. [29]; however, Li et al. [30] achieved an encouraging result for DCR and PD rates. Therefore, we propose that more convincing high-quality research is needed to identify the effectiveness of PD-1 inhibitors in combination with regorafenib in patients with pMMR-MSI-L mCRC.

Furthermore, only 3 studies chose PD-L1 instead of PD-1 inhibitors, but these 3 studies enrolled a large number of patients (476 patients). We conclude that the effectiveness of PD-1 inhibitors may be better than that of PD-L1 inhibitors, although the meta-regression analysis shows that we cannot consider this factor as the source of heterogeneity. We postulate that this result is associated with confounding bias. The MMR-MSI status of all 3 studies in the PD-L1 subgroup was dMMR-MSI-H < 5%, which led to worse effectiveness. In the 11 studies using PD-1 inhibitors, the differences in the effectiveness indicators of nivolumab and pembrolizumab were not significant. For the assessment of the safety of PD-1/PD-L1 inhibitors, we chose AEs and SAEs as representative measures. We found that the AEs and SAEs of monotherapy and PD-1 inhibitors were lower than those of combination therapy and PD-L1 inhibitors, although the meta-regression analysis showed that they were generally not the cause of heterogeneity.

The median follow-up time of most studies included in our meta-analysis was approximately 1 year, and thus, in some studies, PFS and OS were yet not achieved. Some papers did not report relevant data. For these papers, we sent emails to seek useful data. Unfortunately, we could not obtain sufficient data to analyze the PFS and OS by month.

During the search process, we identified some relevant ongoing high-quality clinical trials. We did not include these studies in our analysis because of the lack of sufficient results. However, these studies will also provide useful data on this topic, and we should continue to monitor them.

This meta-analysis has some limitations. First, the dose and frequency of PD-1/PD-L1 inhibitors varied substantially in different studies, which might have affected the effectiveness and safety of the inhibitors. In addition, when we conducted the analysis of monotherapy vs. combination therapy, PD-1/PD-L1 inhibitors were combined with different drugs with different usages. These variables all may be responsible for the heterogeneity of the included studies, and we were unable to analyze these factors because of the diversity of interventions, as mentioned above. Despite the large heterogeneity in this meta-analysis, we believe that it is meaningful to conduct this meta-analysis at this time and to provide more possibilities for the diagnosis and treatment of colorectal cancer.

## Conclusions

In summary, our meta-analysis concluded that PD-1/PD-L1 inhibitors potentially achieved a positive effect on treating dMMR-MSI-H mCRC. PD-1/PD-L1 inhibitors have widely changed the therapeutic situation of mCRC, including pMMR-MSI-L mCRC. Therefore, further multi-center prospective studies are expected to provide additional insights.

## Supplementary Information

**Additional file 1: Table S1**, **Table S2**, **Table S3**, **Table S4**, **Table S5**, and **Fig S1**, **Fig S2** are shown in the file “supporting information.

## Data Availability

The datasets used and/or analyzed during the current study are available from the corresponding author on reasonable request.

## Abbreviations

CRC: Colorectal cancer
mCRC: Metastatic colorectal cancer
PD-1: Programmed death 1
PD-L1: Programmed cell death 1 ligand 1
dMMR: Mismatch repair deficiency
pMMR: Mismatch repair proficiency
MSI-H: High levels of microsatellite instability
MSI-L: Low levels of microsatellite instability
ICIs: Immune checkpoint inhibitors
PROSPERO: International Prospective Register of Systematic Reviews
ORR: Objective response rate
DCR: Disease control rate
CR: Complete response
PR: Partial response
SD: Stable disease
PD: Progression of disease
AEs: Adverse events
SAEs: Severe adverse events
OS: One-year overall survival rate
PFS: One-year progression-free survival rate
RECIST: Response Evaluation Criteria In Solid Tumors
