# Supplementary material for: Clinical benefits of PD-1/PD-L1 inhibitors in patients with metastatic colorectal cancer: a systematic review and meta-analysis
Source: World J Surg Oncol. 2022 Mar 24;20:93. doi: 10.1186/s12957-022-02549-7 (PMC8944161; doi:10.1186/s12957-022-02549-7)
Supplement: Supplementary file 1 — Additional file 1: Table S1, Table S2, Table S3, Table S4, Table S5, and Fig S1, Fig S2 are shown in the file “supporting information. [file 12957_2022_2549_MOESM1_ESM.docx]

**Supporting information**

**Clinical benefits of PD-1/PD-L1 inhibitors in patients with metastatic colorectal cancer: A systematic review and meta‑analysis**

Xiao Zhang^1,δ^, Zhengyang Yang^1, δ^, Yongbo An^1^,Yishan Liu^1^, Qi Wei^1^, Fengming Xu^1^, Hongwei Yao^1,*^, Zhongtao Zhang^1,*^

^1^Department of General Surgery, Beijing Friendship Hospital, Capital Medical University, 95 Yong-an Rd, Xi-Cheng District, Beijing, China.

^δ^Xiao Zhang and Zhengyang Yang contributed equally to this work.

**^*^Corresponding authors.**

Prof. Hongwei Yao, MD, (E-mail: yaohongwei@ccmu.edu.cn)

Prof. Zhongtao Zhang MD, (E-mail: zhangzht@ccmu.edu.cn)

**Table S1. Egger test of included studies.**

| indicator | P | number of articles |
| --- | --- | --- |
| ORR | 0.92 | 14 |
| DCR | 0.6 | 14 |
| CR | 0.756 | 14 |
| PR | 0.884 | 14 |
| SD | 0.135 | 14 |
| PD | 0.725 | 14 |
| AEs | 0.279 | 14 |
| SAEs | 0.303 | 14 |
| PFS | 0.085 | 9 |
| OS | 0.615 | 7 |

P value < 0.05 means significant statistical differences. objective response rate (ORR), disease control rate (DCR), complete response rate (CR), partial response rate (PR), stable disease rate (SD), progression disease rate (PD), adverse events (AEs), severe adverse events (SAEs), one-year progression-free survival rate (PFS), one-year overall survival rate (OS).

**Table S2. Response rate - monotherapy versus combination therapy subgroup analysis.**

|  | Monotherapy | | |  |  | Combination therapy | | | |  |
| --- | --- | --- | --- | --- | --- | --- | --- | --- | --- | --- |
|  | number | | rate | 95%CI | I^2^% | number | | rate | 95%CI | I^2^% |
| CR | 26 | 0.02 | | 0.00-0.07 | 78.67 | 15 | 0.01 | | 0.00-0.04 | 79.36 |
| PR | 113 | 0.17 | | 0.07-0.31 | 96.29 | 106 | 0.11 | | 0.01-0.27 | 95.87 |
| SD | 111 | 0.22 | | 0.18-0.27 | 18.81 | 173 | 0.29 | | 0.22-0.38 | 74.02 |
| PD | 207 | 0.45 | | 0.31-0.59 | 89.06 | 306 | 0.45 | | 0.24-0.67 | 96.33 |
| ORR | 139 | 0.2 | | 0.07-0.37 | 94.15 | 121 | 0.12 | | 0.01-0.31 | 96.73 |
| DCR | 250 | 0.45 | | 0.29-0.62 | 92.26 | 294 | 0.51 | | 0.29-0.72 | 96.49 |

Complete response rate (CR), partial response rate (PR), stable disease rate (SD), progression disease rate (PD), objective response rate (ORR), disease control rate (DCR).

**Table S3. Response rate - PD-1 versus PD-L1 therapy subgroup analysis.**

|  | PD-1 |  |  |  | PD-L1 |  |  |  |
| --- | --- | --- | --- | --- | --- | --- | --- | --- |
|  | number | rate | 95%CI | I^2^% | number | rate | 95%CI | I^2^%* |
| CR | 41 | 0.03 | 0.01-0.07 | 65.81 | 0 | 0 | 0-0 | - |
| PR | 204 | 0.2 | 0.11-0.31 | 88.19 | 15 | 0.03 | 0.01-0.07 | - |
| SD | 179 | 0.29 | 0.22-0.37 | 71.62 | 105 | 0.22 | 0.18-0.26 | - |
| PD | 198 | 0.36 | 0.24-0.49 | 89.54 | 315 | 0.67 | 0.57-0.77 | - |
| ORR | 245 | 0.23 | 0.12-0.36 | 91.12 | 15 | 0.03 | 0.01-0.07 | - |
| DCR | 424 | 0.59 | 0.45-0.71 | 89.79 | 120 | 0.25 | 0.21-0.29 | - |

*: I^2^ can’t be calculated because the number of studies is 3 or less than 3.

Complete response rate (CR), partial response rate (PR), stable disease rate (SD), progression disease rate (PD), objective response rate (ORR), disease control rate (DCR).

**Table S4. Subgroup analysis on adverse events.**

|  | dMMR-MSI < 5% | | | | | | | | |  | | |  | | | dMMR-MSI ≥ 5% | | |
| --- | --- | --- | --- | --- | --- | --- | --- | --- | --- | --- | --- | --- | --- | --- | --- | --- | --- | --- |
|  | | | No. | | rate | | | 95%CI | | I^2^% | | | No. | | rate | | 95%CI | I^2^% |
| AEs | | | 8 | | 0.78 | | | 0.70-0.85 | | 77.62 | | | 6 | | 0.87 | | 0.70-0.98 | 94.19 |
| SAEs | | | 8 | | 0.25 | | | 0.18-0.32 | | 70.84 | | | 6 | | 0.34 | | 0.19-0.51 | 91.74 |
|  | | monotherapy | | | | |  | |  | | combination therapy | | | | | | |  |
|  | | | No. | rate | | 95%CI | | | I^2^% | | | No. | | rate | | | 95%CI | I^2^% |
| AEs | | | 6 | 0.76 | | 0.62-0.88 | | | 90.51 | | | 9 | | 0.9 | | | 0.77-0.98 | 93.98 |
| SAEs | | | 6 | 0.21 | | 0.14-0.29 | | | 73.8 | | | 9 | | 0.38 | | | 0.25-0.53 | 91.27 |
|  | | | PD-1 | | |  | | |  | | | PD-L1 | | | | |  |  |
|  | | | No. | rate | | 95%CI | | | I^2^% | | | No. | | rate | | | 95%CI | I^2^%* |
| AEs | | | 11 | 0.76 | | 0.66-0.86 | | | 86.86 | | | 3 | | 0.98 | | | 0.95-1.00 | - |
| SAEs | | | 11 | 0.23 | | 0.15-0.32 | | | 80.79 | | | 3 | | 0.52 | | | 0.42-0.62 | - |

*: I^2^ can’t be calculated because the number of studies is 3 or less than 3.

No.: the number of studies in the corresponding subgroup.

Adverse events (AEs), severe adverse events (SAEs).

**Table S5. meta-analysis of PD-1 inhibitors.**

|  | nivolumab | |  | pembrolizumab | | | other | |  |
| --- | --- | --- | --- | --- | --- | --- | --- | --- | --- |
|  | rate | 95%CI | I^2^% | rate | 95%CI | I^2^% | rate | 95%CI | I^2^%* |
| CR | 0.05 | 0.00-0.13 | 80.35 | 0.04 | 0.00-0.10 | 69.33 | 0 | 0.00-0.04 | - |
| PR | 0.4 | 0.29-0.51 | 67.31 | 0.21 | 0.11-0.33 | 77.07 | 0 | 0.00-0.04 | - |
| SD | 0.31 | 0.25-0.36 | 0 | 0.21 | 0.16-0.25 | 0 | 0.44 | 0.10-0.82 | - |
| PD | 0.19 | 0.07-0.34 | 86.65 | 0.43 | 0.30-0.56 | 77.34 | 0.56 | 0.18-0.90 | - |
| ORR | 0.46 | 0.33-0.60 | 78.37 | 0.24 | 0.11-0.41 | 87.92 | 0 | 0.00-0.04 | - |
| DCR | 0.77 | 0.63-0.89 | 83.83 | 0.47 | 0.32-0.63 | 84.47 | 0.44 | 0.10-0.82 | - |
| AEs | 0.84 | 0.69-0.95 | 86.71 | 0.72 | 0.53-0.88 | 90.69 | 0.7 | 0.29-099 | - |
| SAEs | 0.28 | 0.22-0.34 | 8.36 | 0.18 | 0.09-0.30 | 77.31 | 0.29 | 0.00-0.83 | - |

*: I^2^ can’t be calculated because the number of studies is 3 or less than 3.

Complete response rate (CR), partial response rate (PR), stable disease rate (SD), progression disease rate (PD), objective response rate (ORR), disease control rate (DCR), adverse events (AEs), severe adverse events (SAEs).

**
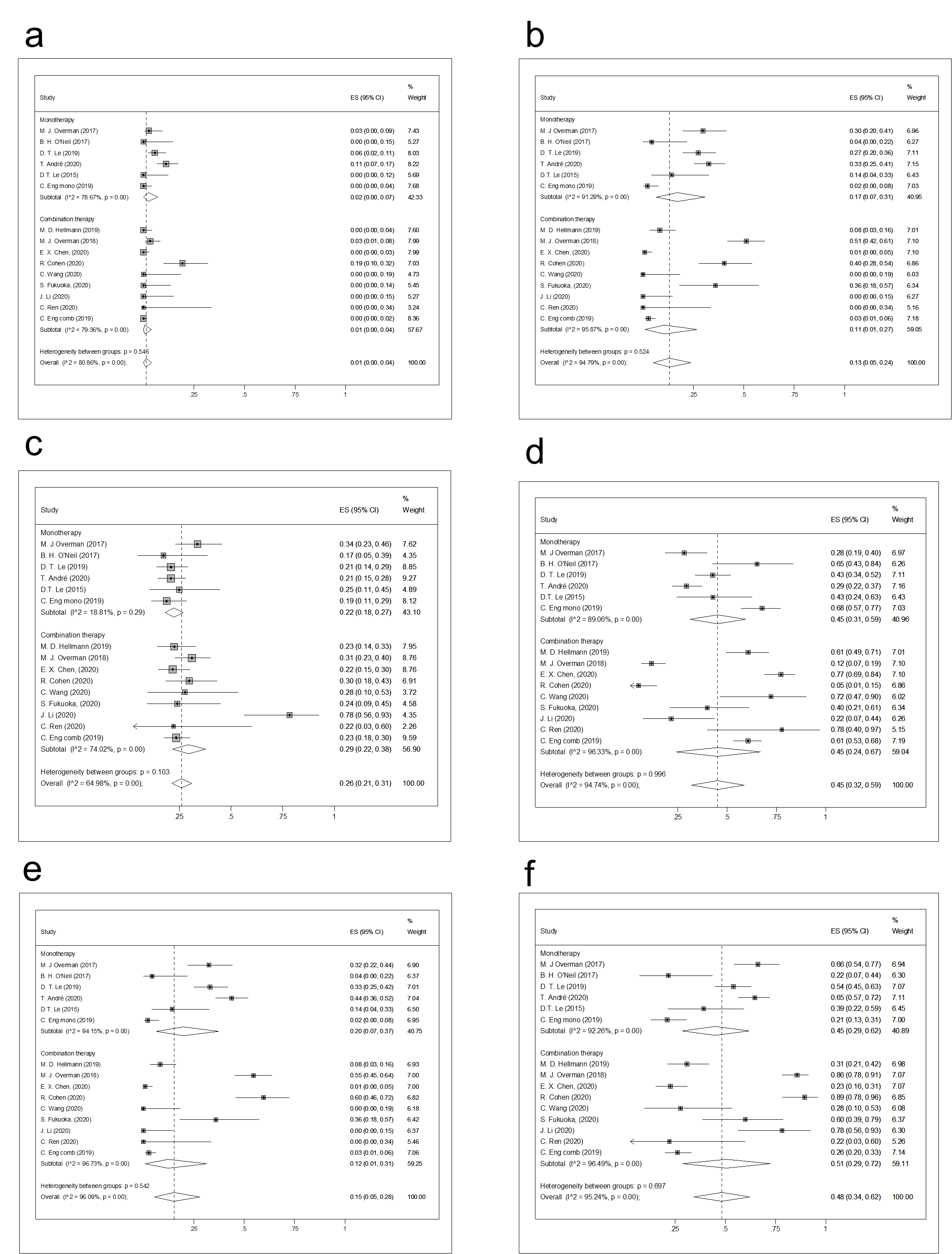
**

**Fig S1. The forest figure of response rate (CR, PR, SD, PD, ORR, DCR) on** monotherapy versus combination therapy subgroup analysis. (a) CR rate on monotherapy versus combination therapy subgroup analysis. (b) PR rate on monotherapy versus combination therapy subgroup analysis. (c) SD rate on monotherapy versus combination therapy subgroup analysis. (d) PD rate on monotherapy versus combination therapy subgroup analysis. (e) ORR rate on monotherapy versus combination therapy subgroup analysis. (f) DCR rate on monotherapy versus combination therapy subgroup analysis. Complete response rate (CR), partial response rate (PR), stable disease rate (SD), progression disease rate (PD), Objective response rate (ORR), disease control rate (DCR).

**
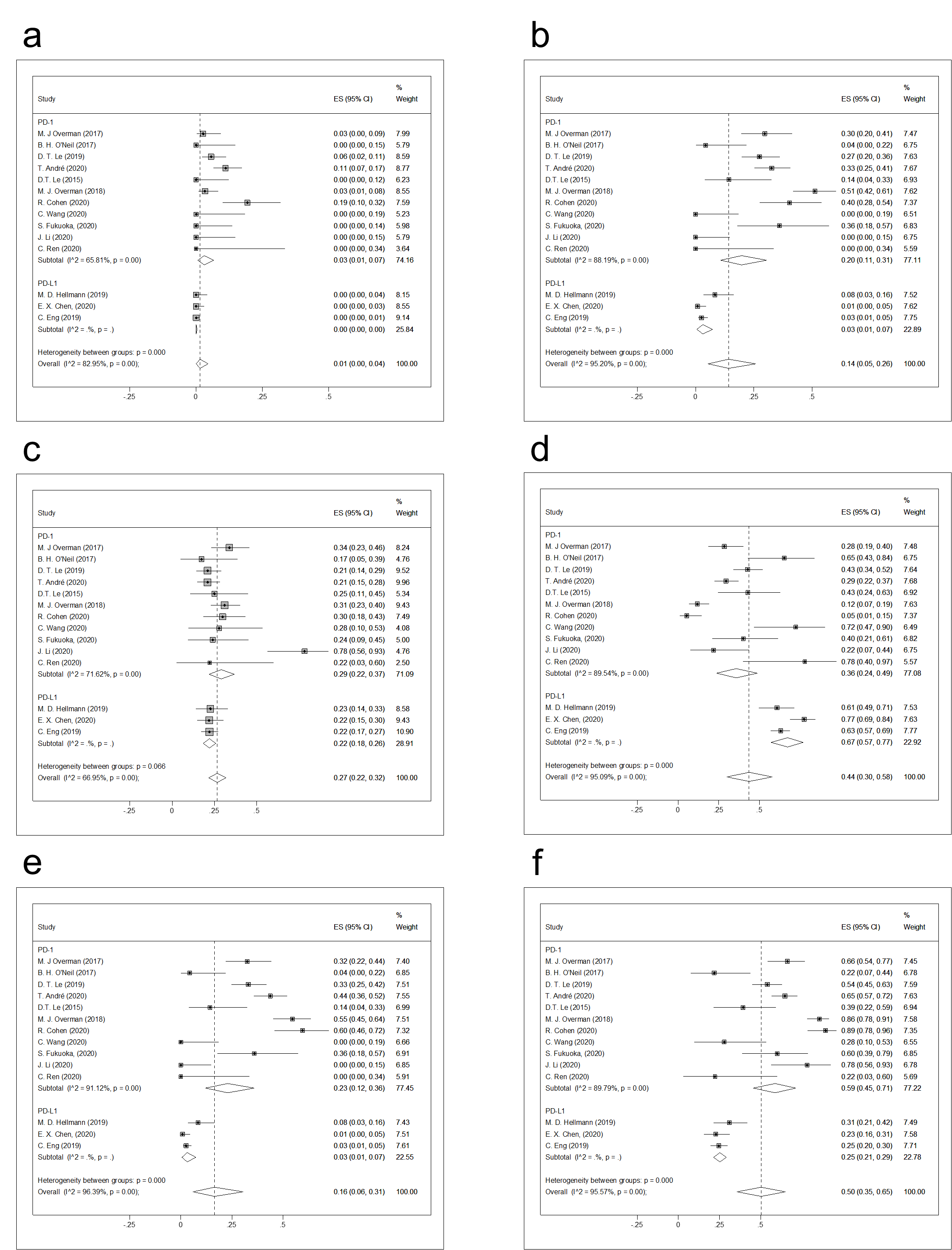
**

**Fig S2. The forest figure of response rate (CR, PR, SD, PD, ORR, DCR) on** **PD-1 inhibitors versus PD-L1 inhibitors subgroup analysis.** (a) CR rate on PD-1 inhibitors versus PD-L1 inhibitors subgroup analysis. (b) PR rate on PD-1 inhibitors versus PD-L1 inhibitors subgroup analysis. (c) SD rate on PD-1 inhibitors versus PD-L1 inhibitors subgroup analysis. (d) PD rate on PD-1 inhibitors versus PD-L1 inhibitors subgroup analysis. (e) ORR rate on PD-1 inhibitors versus PD-L1 inhibitors subgroup analysis. (f) DCR rate on PD-1 inhibitors versus PD-L1 inhibitors subgroup analysis. Complete response rate (CR), partial response rate (PR), stable disease rate (SD), progression disease rate (PD), Objective response rate (ORR), disease control rate (DCR).
